# Mental health provider and youth service users’ perspectives regarding implementation of a digital mental health platform for youth: A survey study

**DOI:** 10.1177/20552076241289179

**Published:** 2024-10-14

**Authors:** Julia Hews-Girard, Katherine Bright, Marianne Barker, Emily M Bassi, Frank Iorfino, Haley M LaMonica, Karen Moskovic, Melanie Fersovitch, Leanne Stamp, Jessica Gondziola, Ian Hickie, Gina Dimitropoulos

**Affiliations:** 1 70403 Faculty of Nursing, 2129 University of Calgary, Calgary, Canada; 2 Faculty of Social Work, 2129 University of Calgary, Calgary, Canada; 3 Alberta Children's Hospital Research Institute, 2129 University of Calgary, Calgary, Canada; 4 651991 Faculty of Nursing and Midwifery, Mount Royal University, Calgary, Canada; 5 Brain and Mind Center, 4334 University of Sydney, Sydney, Australia; 6 3146 Alberta Health Services, Calgary, Canada; 7 Mathison Center for Mental Health Research and Education, 2129 University of Calgary, Calgary, Canada

**Keywords:** Digital mental health, CFIR, youth mental health, implementation, mental health

## Abstract

**Background:**

For youth and young adults (YYAs) with mental health concerns, digital mental health (dMH) can improve access to care and support collaboration with providers. Measurement-based care using a dMH platform may further optimize YYA outcomes by individualizing treatment approaches. Engaging service providers and YYA provides an opportunity to better understand how to mitigate implementation challenges.

**Aim:**

Explore the experiences of mental health care providers and YYAs regarding the implementation of a dMH platform for YYAs accessing mental health care in multiple care settings.

**Methods:**

Mental health care providers and YYA service users completed an electronic survey exploring their experiences and perceptions of implementing a dMH platform. Survey design, data analysis, and reporting were informed by the Consolidated Framework for Implementation Research (CFIR).

**Results:**

A total of 195 individuals (100 providers, 95 YYAs) responded. Of those, 48 providers and 79 YYAs reported using the platform at least once. Both groups identified several important factors supporting implementation including collaborative relationships between providers and YYAs, the ability to monitor mental health outcomes, and increased YYA engagement in care. The need for increased provider training and automatic reminders for YYAs to use the platform were common barriers. Each group perceived the other to be uninterested in using the platform, highlighting the importance of using all stakeholder views to inform implementation planning.

**Conclusions:**

Successful implementation of dMH for care of YYA requires ongoing, user-informed training, integration into existing workflows, and alignment with YYA priorities for care. Future work exploring provider and youth perceptions of the others’ “buy-in” is needed to inform future implementation strategies.

## Introduction

Mental illness in youth and young adults (YYA) aged 12–25 years is a global public health concern.^1,2^ Youth and young adulthood is a critical period of developmental and transitional change,^3,4^ and it is estimated that one in five youth experiences a mental health concern each year.^5^ In a recent epidemiological meta-analysis, the authors reported that many mental health disorders have an onset occurring before ages 14, 18, and 25 in 33%, 48.4%, and 62.5% of individuals, respectively.^6^ Concerningly, most youth will remain undiagnosed/untreated in part due to barriers to accessing mental health services.^5^ Common barriers include lack of availability/accessibility of appropriate treatment options, service cost, youth's lack of knowledge about mental health care systems, confidentiality issues, perceived stigma of mental health conditions, and youth's geographical location.^7^ Left untreated, YYA with significant mental health concerns are more vulnerable to subsequent stressors and impairment in functioning across the lifespan.^8–10^

Isolation and increased stress due to the COVID-19 pandemic have further contributed to worsening mental health among YYA.^11–13^ The abrupt disruptions to educational routines and social interactions contributed to increased feelings of uncertainty and anxiety^14,15^ and tended to exacerbate existing mental health issues such as depression and anxiety.^14^ The loss of traditional support systems, coupled with the economic repercussions of the pandemic, has heightened stress levels for individuals and their families^16^ and highlighted the need for targeted mental health interventions to aid YYA in navigating these psychological issues.^17^

### Digital mental health and measurement-based care

Digital Mental Health (dMH) is an emerging care modality to addressing the specific needs of youth.^18^ dMH services cater to the preferences of tech-savvy YYA, providing convenient and confidential avenues to seek support including apps, e-tools, crisis resources, and cues to discuss care options with their clinicians.^19–21^ A dMH platform can foster a sense of connection, making this a promising avenue to reach and engage youth.^20,22,23^ Prior work has reported that providers are supportive of dMH, particularly regarding the ability to provide extra support to clients and potential for extra treatment options and provision of general education.^21,24,25^ In contrast, two recent (2019, 2020) systematic reviews found that unrelatable (e.g. generic) educational material/content was not appealing to youth, and they were more likely to stop using the intervention in those situations.^20,26^ While studies have reported on the gaps between the potential positive impact of digital mental health interventions and actual uptake,^24^ they have focused on perceptions and experiences of providers, organizations, and funders.^24,27^ Reporting of YYA perceptions of this gap, as well as the implementation process in general, remains limited.

dMH is grounded in measurement-based care (MBC), shared decision making, personalized medicine, and tailored interventions.^28,29^ MBC involves the systematic use of standardized assessments to gauge and monitor a person's mental health status over time.^30,31^ MBC allows providers to gain a comprehensive understanding of changes in a patient's symptoms and functioning over time,^32^ supports timely intervention and adjustment of treatment plans,^33^ increases patient engagement with care,^34^ and is associated with improved patient outcomes.^34,35^ Evaluation of MBC in youth mental health care settings has thus far focused on the perceptions and experiences of providers rather than YYAs^36^; inclusion of the youth’s voice is important to understand how dMH and MBC work in a real-world setting.

Multiple challenges in the implementation, uptake, and integration of dMH into current workflow processes within clinical services have been noted.^37^ Much of the literature has focused on clinician perspectives of implementation issues including digital literacy and social and organizational factors impacting workflow integration and clinician uptake.^38–41^ The literature exploring the YYA perspectives and recommendations regarding dMH is sparse. Additionally, recommendations that reflect both YYA's and mental health care providers’ experiences are lacking.^42^ Understanding the perspectives of clinicians and service users is vital for effective planning, resource allocation, community partner engagement, sustainability, customization, and continuous improvement of dMH.^23^

### Consolidated framework for implementation research

The Consolidated Framework for Implementation Research (CFIR) is a conceptual framework used to identify factors that impact implementation and effectiveness of interventions and/or programs.^43^ The CFIR includes 39 constructs across five domains associated with implementation outcomes: intervention characteristics (e.g. stakeholder perceptions of complexity of the intervention), outer setting (e.g. policy), inner setting (e.g. organizational features), individuals (e.g. knowledge of the intervention), and implementation process (e.g. reflections on the process). The CFIR has been used extensively to guide the evaluation of both the intervention and the implementation process in a wide variety of health care settings^36,44,45^ and implementation/evaluation approaches (including learning health systems and rapid cycle evaluations.^36,45^ An important outcome of using the CFIR framework is the ability to identify practical, actionable findings that can be used to guide the implementation process.^45^

### Current study

Systematic evaluation of the implementation of dMH interventions is key to optimizing patient and system outcomes. Therefore, this study aimed to understand the experiences of service providers and YYAs regarding the implementation of a dMH platform in youth mental health settings and develop recommendations for future implementation activities. To achieve this, a comprehensive survey was conducted, focusing on identifying the barriers and facilitators that influence the adoption and use of dMH. This survey was structured around the CFIR which provided a framework for developing survey questions, guiding data analysis, and organizing the reporting of results. The multi-faceted approach of the CFIR model allowed for an in-depth exploration of the five domains including intervention characteristics, inner and outer settings, characteristics of individuals, and the process of implementation.

## Methods

The present study is part of a larger study exploring implementation of dMH into community-based and specialized mental health care settings.^21^ The study methods have been previously described^21^; briefly a dMH platform was implemented in several mental health settings throughout Alberta, and subsequently evaluated to examine the impact of dMH on clinical (patient) and health system outcomes. The present study used a survey design to collect information regarding barriers and facilitators of implementing dMH from the perspective of providers and youth. All providers who received training and were onboarded to the platform in all settings and all YYA who consented and accepted the invitation to use the dMH platform between January 2023 and April 2024 were invited to participate in the survey.

### Study setting and participants

The dMH platform was implemented in four mental health service settings across Alberta including primary care networks (PCNs), schools, specialized mental health services, and community mental health centers.^21^ Potential participants included mental health care providers (e.g. counsellors) and managers employed in one of the included mental health service settings and youth aged 15–24 years who accessed care from these providers. Additional inclusion criteria for providers included completion of training and onboarding requirements for the dMH platform. Additional inclusion criteria for youth included: ability to read and understand English, access to a computer or mobile device and internet. Given that the aim of the work was to explore experiences of implementing dMH among individuals who had been invited/asked to use it, no sample size was calculated.

### The platform

The dMH platform used for this work was the Innowell platform. The platform details – including adaptations for the larger project - have been previously described.^21,46^ In brief, Innowell is designed to enhance mental health clinical care and support and to facilitate a more personalized and collaborative approach to mental health care.^46^ Implementation into the service settings involved in the work described in this paper was supported by a clinical implementation team.

### CFIR application

This work was divided into two stages: survey design and data collection and analysis. The CFIR model served as a framework for structuring survey questions, data analysis, and result reporting. The CFIR model is freely available for use in research.^43,44^

#### Survey design and data collection

The survey was created for this project by the research team; members have expertise in implementation science, program evaluation, mental health, and measurement-based care. Closed and open-ended questions, as well as Likert-scale structured questions, were used to collect data assessing barriers and facilitators of implementation of dMH from providers and youth who did, and did not, use the platform. Questions were structured around four CFIR domains—intervention characteristics (e.g. complexity of the intervention). inner setting (characteristics of the delivery sites for the platform), individuals (e.g. roles and beliefs of the individuals using the platform), and implementation process.^43^ Separate surveys with similar questions were developed for each user group (providers and YYAs); wording of questions changed to reflect the intended participant (e.g. “pick the option that best describes where your organization is located” became “pick the option that best describes where you live”). The survey was pretested by team members not involved in development to ensure clarity, comprehensiveness, and appropriateness. Table 1 provides examples of survey questions for each of the domains by participant group; the full survey is included in Supplemental Table 1.

**Table 1.** CFIR domains and examples of associated questions.

CFIR domain	CFIR guiding questions*	Type of question	Study examples
Intervention characteristics	What will make the platform work in your setting? How complicated is the platform to use?	Closed-ended (yes/no)	*Providers:* *Which of the following might help you integrate [the platform] into your everyday work?* *Frequency of use of the platform.* *Youth:* *Frequency of use of the platform.* *Time spent on the platform*
Inner setting	Did you have support to implement the platform? What level of support from management? (providers) What level of support from clients? (providers) What level of support from providers? (youth)	Closed-ended (multi-choice; yes/no)	*Providers:* *Is lack of support from leadership in the organization a barrier to implementation?* *My organization uses a strategy of training to implement the [the platform].* *Youth:* *I feel my provider is interested in using the platform with me* *More training from my provider about how to use the platform would be helpful.*
Individuals	What are your beliefs and knowledge about dMH and/or the platform? What is your comfort with dMH? How long have you been in the role? (providers)	Closed-ended (multi-choice; yes/no)	*Providers:* *Years in the role?* *Do you believe the platform increases engagement with youth?* *Frequency of use of the platform* *Youth:* *I feel motivated to use the questionnaires* *Age* *Employment status*
Implementation Process	What is the goal of implementation? Why was the platform implemented? Where was it implemented and was it easy to implement?	Closed-ended (multi-choice; yes/no)	*Providers:* *The [platform] meets the needs of my clients.* *The [platform] aids in providing comprehensive care* *Youth:* *I have a good idea of when to use the platform* *The apps or e-tools are easy for me to use*

*CFIR: Consolidated Framework for Implementation Research; adapted from Damschroder et al., 2020.

Data was collected via RedCap (v.13.7.31; Vanderbilt University, 2024). All providers working in the study sites were emailed an invitation to participate in the provider survey three months following completion of initial training and onboarding. Two reminders were sent one and two weeks following the initial invitation. If the survey remained incomplete following the second reminder, the individual was excluded. Given the pragmatic design, potential youth participants were recruited using several steps: providers discussed the dMH platform and study with all eligible youth and provided a registration link for the platform to those who requested it. Youth who subsequently registered to use the platform and consented to be contacted for research were sent a link to the youth survey. As for the providers, two reminders were sent one and two weeks following the initial invitation. If the survey remained incomplete following the second reminder, the individual was excluded. Survey data for both groups was collected from February 2023 to May 2024 inclusive.

#### Data analysis and reporting

Univariate descriptive statistics (e.g. frequency, percent) were used to summarize demographic data and responses to both closed-ended and Likert scale questions. Responses to open-ended questions were analyzed deductively and similar comments combined and reported with the frequency of occurrence in the data. All responses were categorized according to the four guiding CFIR domains and checked for relevance to the final domain (e.g. outer setting). This approach was repeated for each participant group separately to allow for comparison and identification of similar barriers and/or facilitators, as well as potential solutions/recommendations. Demographic data was analyzed for all individuals who replied to the survey. Assessment of implementation was restricted to individuals who reported using the platform. Non-use included individuals who did not use the platform for any reason. Population centers were defined according to Statistics Canada (2022): small (population between 1000 and 29,999), medium (30,000–100,000), and large (>100,000).^47^

### Ethics

The study was approved by the University of Calgary's Conjoint Health Research Ethics Board (CHREB) (REB 20-1137). Participants provided informed consent prior to completing the survey. An electronic consent form was included as the first page of the survey; consent to participate was indicated by choosing “yes, I consent to participate” and continuing to the survey.

## Results

Overall, 253 individuals were invited to participate—131(51.8%) providers, 122 (48.2%) YYA. The total response rate was 77.1% (195/253) (providers: 76.3% (100/131), YYA: 77.9% (95/122)). The focus of this work is on implementation factors requiring consideration during implementation and the associated contexts (e.g. length of time a provider has been in the role; if a YYA is a student) that are important for understanding the experiences underpinning these.

### Overall participant demographics

*Providers:* A total of 100 providers responded. Of those, 82 (82.0%) were female, 30 (30%) were between 30 and 39 years of age, 92 (92.0%) identified English as their primary language, and 49 (49.0%) were of European origin (Table 2). Most (40/100; 40.0%) providers were social workers. Most respondents worked in specialized mental health services (58/100; 58.0%) or school districts (25/100; 25.0%) and most reported being in their current role and/or with their current organization for ≤5 years (66/100, 66.0%; 55/100, 55.0%, respectively). Most providers (79/100; 79.0%) worked full-time and in small population centers (63/100; 63.0%). 48/100 (48.0%) reported using the platform with youth, 28/100 (28%) reported not inviting youth to use the platform, and 24/100 (24%) reported inviting youth, but not having had the opportunity to use the platform.

**Table 2.** Participant demographics.

	Providers (*n* = 100)		Youth & young adults (*n* = 95)
Age (years)		Age (years)	
<30	21 (21.0)	15–17	28 (29.5)
30–39	30 (30.0)	≥18	67 (70.5)
40–49	26 (26.0)		
>50	24 (24.0)		
Gender		Gender	
Female	82 (82.0)	Female	73 (76.8)
Male	14 (14.0)	Male	14 (14.7)
Prefer not to say	5 (5.0)	Prefer not to say	7 (7.4)
Primary language		Primary language	
English	92 (92.0)	English	86 (90.5)
Other/prefer not to answer	9 (9.0)	Other/prefer not to answer	8 (9.5)
Ethnicity		Ethnicity	
European	49 (49.0)	European	44 (46.3)
Multiple ethnicities	12 (12.0)	Multiple ethnicities	18 (18.9)
Indigenous/African	8 (8.0)	Indigenous/African	7 (7.4)
Asian/Caribbean	5 (5.0)	Asian/Oceania	12 (12.6)
Other North American*	8 (5.0)	Other North American*	5 (5.3)
Prefer not to say/unsure	18 (18.0)	Prefer not to say/unsure	6 (6.3)
Community		Community	
Large population center	19 (19.0)	Large population center	43 (45.3)
Medium population center	19 (19.0)	Medium population center	8 (8.4)
Small population center	63 (63.0)	Small population center	44 (46.3)
Professional designation		Employment status	
Social work	40 (40.0)	Student	57 (60.0)
Psychology	13 (13.0)	Employed	88 (92.6)
Counsellor/Therapist	25 (25.0)		
Other**	23 (23.0)		
Place of work			
Specialized Mental Health	58 (58.0)		
School	25 (25.0)		
Primacy Care Network	12 (12.0)		
Other***	6 (6.0)		

Note: demographics are for all individuals who consented to, and filled out, the survey; all values are *n* (%) unless otherwise noted; large population center: population >100,000; medium population center: population between 30,000 and 99,999; small population center: population between 1000 and 29,999); *other NA includes: Acadian, American, Quebecois; **other includes: nursing, teacher/educator, administration, other, missing; ***other includes community-based setting, missing, other.

*YYA:* A total of 95 YYAs responded. Of those, most were ≥18 years of age (67/95; 70.5%), female (73/95; 76.8%), students (57/95; 60.0%), and employed at least part-time (88/95; 92.6%). Most reported English as their primary language (86/95; 90.5%) and being of European origin (44/95; 46.3%). Most lived in small (44/95; 46.3%) or large population centers (43/95; 45.3%). Among YYAs, 79 (83.2%) reported using the platform.

### Implementation assessment

#### Intervention characteristics.

*Providers:* Of the 48 providers who reported using the platform, 30 (62.5%) reported using the platform in less than half of sessions, and 15 (31.3%) reported using it in more than half (Table 3). The applications (apps) and resources were perceived to be helpful for client self-management by 21/48 (43.8%) and 19/48 (39.6%) of providers respectively.

**Table 3.** Assessment of implementation.

CFIR domain & questions	Providers (*n* = 48)	CFIR domain & questions	Youth & young adults (*n* = 79)
**Intervention characteristics**			
Frequency of use in sessions		Frequency of use	
* Every session*	6 (12.5)	* Once a month*	42 (53.2)
* More than half of sessions*	9 (18.8)	* 2 to 10 times a month*	37 (46.9)
* Less than half of sessions*	30 (62.5)	Length of time logged on	
* Missing/never/other*	*unable to report*	* 0–15 min*	38 (48.1)
		* 16–30 min*	33 (41.8)
		* 31–60 min*	8 (10.1)
The apps in the platform are helpful for self-management by clients^A^	21 (43.8)	The apps in the platform are helpful for self-management^C^	24 (30.4)
The resources in the platform are helpful for self-management by clients^A^	19 (39.6)	Frequency of updating surveys	
		* As needed*	26 (32.9)
		* Not very often/Never*	34 (43.0)
		* Every session/more than every session*	18 (22.8)
**Inner setting**			
Degree of support received from: management		Perceived support from provider*	
* Strong*	36 (75.0)	* My provider is interested in using the platform with me*	14 (17.7)
* Somewhat/Neutral*	12 (25.0)	* Reviewing results with my provider is helpful*	53 (67.1)
organization		* Reviewing results in sessions*	15 (19.0)
* Strong*	32 (66.7)	* Follow-up by provider regarding concerning results*	31 (39.2)
* Somewhat/Neutral*	16 (33.3)		
Need for more^A^:		Need for more*:	
* time to learn/practice*	6 (12.5)	* training from my provider*	6 (7.6)
* youth understanding of the platform*	25 (52.1)	* better communication from my provider regarding the platform*	18 (22.8)
* supports/service roles*	12 (25.0)	* text/emails reminders*	45 (57.0)
**Individuals**			
Years in role		Age	
≤*5 years*	28 (58.3)	* 15–17 years*	24 (30.4)
≥*6 years*	20 (41.7)	* 18–25 years*	54 (68.4)
Years in organization		Employment status	33 (41.8)
≤*5 years*	27 (56.3)	* No*	40 (50.6)
≥*6 years*	21 (43.8)	* Yes*	6 (7.6)
		* Missing/other*	
Comfort with the platform		Comfort with platform^A^	
* Percentage of caseload introduced to the platform*		* I feel motivated to use the surveys*	37 (46.8)
* 1–49%*	37 (77.1)	* I feel motivated to use the apps or e-tools^C^*	12 (28.6)
* 50–100%*	11 (22.9)	* I would refer a friend/family member to the service that offers the platform*	62 (78.5)
* I feel comfortable implementing the platform* ^A^	41 (85.4)		
Individual-level barriers to use*		Individual-level Barriers to Use	
* Lack of personal capacity/interest*	12 (25.0)	* Difficulty understanding how the platform helps my mental health*	9 (11.4)
* Lack of understanding of the platform*	8 (16.7)	* Not seeing the value in the platform*	7 (8.9)
Individual-level facilitators of use*		* Forgetting to use the platform*	58 (73.4)
* Personal skill level*	27 (56.3)		
* Professional confidence (assessments)*	31 (65.8)		
I believe that the platform:*^A^		Individual-level facilitators of use*	
* Helps engage youth*	36 (75.0)	* Understanding the relationship between the platform and mental health*	21 (26.6)
* Creates positive experiences*	28 (58.3)	Viewing results immediately*	64 (81.0)
* Helps build relationships with youth*	22 (45.8)	I felt that using the platform*:	
		* Contributed to improved mental health*	18 (22.8)
		* Helped my provider better understand my mental health*	54 (68.4)
		* Helped me feel more involved in my care*	66 (88.5)
**Implementation process**			
Reasons for implementation*		Reasons for use	
* Informing treatment planning*	26 (54.2)	Good addition to the care I receive*	69 (87.3)
* Outcome monitoring*	34 (70.8)	Helps me and my provider collaborate on my wellbeing*	65 (82.3)
* Encourage proactive mental health care*	40 (83.3)	Helps me track my mental health between sessions*	67 (84.8)
* Monitor at risk youth*	16 (33.3)	* Helps me better understand my mental health*	52 (65.8)
* Build collaborative working relationship with youth*	26 (54.2)		
* Youth/Family interest*	22 (45.8)		
Goal of implementation*^A^			
* Provide better discharge planning*	12 (25.0)		
* Provide more comprehensive care*	25 (52.1)		
* Aids in monitoring progress of clients*	32 (66.7)		
* Meets client needs for care*	22 (45.8)		
Implementation process^A^		Implementation process*	
* Platform was easily implemented during intake*	17 (35.4)	* I have a good idea of when to use the platform*	35 (44.3)
* Platform was easily implemented during treatment planning*	23 (47.9)	* I understand how the platform works*	58 (73.4)
* Platform was difficult to implement during case review* ^B^	32 (66.7)	* I feel like I had a choice of what questions to answer*	65 (82.3)
		* I did not feel pressured to share information*	69 (87.3)
Level of difficulty to implement		Level of difficulty to use*	
* Easy/Very Easy*	15 (31.3)	* Going through the surveys was easy for me*	54 (68.4)
* Neutral*	15 (31.3)	* The apps or e-tools are easy for me to use^C^*	28 (66.7)
* Difficult/Very Difficult*	18 (37.5)		

Note: includes only individuals who had youth on the platform (providers) or who used the platform (YYA); all results are expressed as frequency (%); *respondents could pick >1 option; ^A^number of individuals that agree/strongly agree with the statement; ^B^agree/neutral; ^C^denominator = 42.

*CFIR: Consolidated Framework for Implementation Research; adapted from Damschroder et al., 2020.

*YYA:* Of the 79 YYAs who reported using the platform, most (42/79; 53.2%) reported using the platform once-a-month, while 37/79 (46.9%) used it two to 10 times a month. Most (38/79; 48.1%) YYAs reported using the platform for ≤15 min each time, while 33/79 (41.8%) reported using it for between 16 and 30 min each time. The apps were perceived by 24/79 (30.4%) of YYAs to be helpful for self-management. Most (34/79; 43.0%) reported that they did not update the surveys/questionnaires very often; 18/79 (22.8%) reported updating them at least every session with their provider.

#### Inner setting

*Providers:* Most providers using the platform reported “strong” support from their management (36/48; 75.0%) and organization (32/48; 66.7%) during implementation. Most providers (25/48; 52.1%) reported that better YYA understanding of the platform would be helpful for implementation, as would more support roles (12/48; 25.0%) and time for staff to practice using the platform (6/48; 12.5%).

*YYA:* Among YYAs using the platform, perceived support during implementation was reported in several ways. First, most felt that reviewing results with their provider was helpful (53/79; 67.1%) as was provider follow-up regarding concerning results (31/79; 39.2%). Of those who responded, 15/79 (19.0%) reported having reviewed results in sessions with the provider, and 14/79 (17.7%) felt that the provider was interested in using the platform with them.

#### Individuals

*Providers:* Most providers using the platform had been in their role (28; 58.3%) and organization (27/48; 56.3%) for five or less years. Most (37/48; 77.1%) had introduced the platform to less than 50% of their case load. Over 85% (41/48) of providers using the platform reported feeling comfortable implementing the platform. The top two individual-level barriers to use among providers were lack of personal capacity/interest (12/48; 25.0%) and lack of understanding of the platform (8/48; 16.7%); facilitators were personal skill level (27/48; 56.3%) and professional confidence with assessments (31/48; 65.8%). Providers believed that the platform helped engage youth in care (36/48; 75.0%), created a positive experience for youth (28/48; 58.3%), and helped build relationships with youth (22/48; 45.8%).

*YYA:* The majority of YYA using the platform were between 18 and 25 years old (54/79; 68.4%) and reported being employed (40/79; 50.6%). Of those using the platform, 37/79 (46.8%) reported feeling motivated to use the questionnaires/surveys and 12/79 (28.6%) reported feeling motivated to use the apps/e-tools. The top three individual-level barriers to use were difficulty understanding how the platform would help their mental health (9/79; 11.4%), not seeing value in the platform (7/79; 8.9%), and forgetting to use it (58/79; 73.4%); facilitators were understanding the relationship between the platform and mental health (21/79; 26.6%) and being able to view results immediately (64/79; 81.0%). YYAs believed that the platform contributed to improved mental health (18/79; 22.8%), helped the provider better understand their mental health (54/79; 68.4%), and helped them feel more involved in their care (66/79; 88.5%).

#### Implementation process

*Providers:* Reasons for implementing the platform included informing treatment planning (26/48; 54.2%), monitoring outcomes (34/48; 70.8%), encouraging proactive mental health care (40/48; 83.3%), and building collaborative working relationships with YYAs (26/48; 54.2%). Perceived goals of the implementation included helping to monitor client progress (32/48; 66.7%) and providing more comprehensive care (25/48; 52.1%). Most providers reported that the platform was easily implemented during treatment planning (23/48; 47.9%) compared to during intake or case review. Of providers, 15/48 (31.3%) reported that the platform was overall easy or very easy to implement.

*YYA:* Among YYAs using the platform, reasons for use included being a good addition to their care (69/79; 87.3%), supporting collaboration with their provider (65/79; 82.3%), and tracking mental health between sessions (67/79; 84.8%). Most YYAs reported understanding how the platform works (58/79; 73.4%) and not feeling pressured to share information (69/79; 87.3%). Overall, YYAs reported that the surveys (54/79; 68.4%) and apps (28/79; 66.7%) were easy to use.

### Assessment of non-use

Of the individuals who completed the survey, 52 providers and 16 YYAs reported not using the platform (Supplemental Table 1) (Table 4).

**Table 4.** Assessment of non-users.

	Providers (*n* = 52)		Youth & young adults (*n* = 16)
Reasons for not using the platform		Reason for not using the platform	
* Limited interest as a provider*	7 (13.5)	* Forgot/haven’t heard of it*	6 (37.5)
* Lack of youth interest*	21 (40.4)	* Not interested/not helpful*	6 (37.5)
* Change fatigue*	9 (17.3)		
* Not directly working with youth*	12 (23.1)		
Things that would help with uptake		Things that would help with uptake	
* More information about usefulness to inform treatment planning*	6 (11.5)	* Better understanding of how the platform could help*	6 (37.5)
* Time for providers to use and learn the platform*	15 (28.8)	* If provider reviewed use of platform and/or seemed more interested*	7 (43.8)
* Improved understanding among youth about platform use*	10 (19.2)		

*Providers:* Most reported being in their role and/or organization for five years or less (37/52, 71.2%; 27/52, 51.9% respectively). Of those in their role for ≤5 years, 13/37 (8.1%) reported having no direct client contact but supported other providers who did. Most worked in specialized mental health settings (33/52; 63.5%) and were social workers (21/52; 40.4%). Over 55% (29/52) of providers reported working in small population centers. Aside from not having direct contact with YYAs, the top two reasons for not using the platform were perceived lack of interest from YYAs (21/52; 40.4%) and limited provider interest (7/52; 13.5%). Increased time to learn how to use the platform was identified as a factor that would contribute to increased uptake (15/52; 28.8%).

*YYAs:* Most reported being ≥18 years of age (12/16; 75.0%), employed at least part-time (13/16; 81.3%), and living in a large population center (9/16; 56.3%). Two reasons for not using the platform were identified: forgot/hadn’t heard of it (6/16; 37.5%) and limited interest/didn’t find it helpful (6/16; 37.5%). Increased provider interest in using the platform with YYAs and/or increased review of the platform by providers were identified as factors that would contribute to increased uptake (7/16; 43.8%).

## Discussion

The purpose of this study was to understand the experiences and perceptions of mental health care providers and YYAs regarding the implementation of a dMH platform into several mental health service settings. Overall, we found that providers and YYAs viewed the dMH platform as a positive addition to care, believed that the platform promoted engagement and collaboration between them, and supported monitoring of mental health outcomes. While both groups felt supported in their use of the platform, a need for increased education and training was identified. Both providers and YYAs perceived that the other group was hesitant to use the platform and that this hesitancy created barriers to implementation.

The *intervention, individuals, and implementation* domains of the CFIR address implementation factors related to acceptability and usability of the intervention. Digital health interventions that are easy to use and understand, support individuals in meeting their goals, and align with the beliefs of the individual toward both digital health and general care have been shown to contribute to the acceptability of an intervention.^22,27,48^ These have also been associated with improved uptake of dMH in real-world settings^22,24^ and been shown to contribute to the integration of the intervention into daily routines.^48^ As part of this work, we focused on implementing a digital platform that would address the needs, beliefs, and goals of both YYAs and providers, and ultimately be acceptable for both groups. Our results support these efforts as both YYAs and providers found the dMH platform to be a good addition to usual care and reported that it supported the development of collaborative relationships and contributed to increased YYA engagement in care. Our findings suggest that further work needs to be done to identify where in the clinical workflow the platform is easiest to implement and how that implementation can be optimized moving forward.

The *inner setting* domain addresses implementation factors associated with organizational support; for providers this includes support from managers and the organization.^44^ Limited information exists regarding how organizational support is defined for YYA users of dMH; prior work in adults suggests that organizational support for patients may include provider support to understand medical content and/or support understanding of the platform.^24,49^ Therefore, we defined organizational support for YYAs as support provided by mental health care providers with the purpose of facilitating uptake of the platform. Consistent with prior work,^22,25,49,50^ a need for increased organizational support in the form of education addressing how and when to use the platform was identified in our work. Specifically, providers identified the need for more education and time/opportunities to practice, and YYAs identified the need for providers to review the platform during sessions. Research suggests that these challenges may be addressed for providers using hands-on learning (e.g. “practice time” with the app/platform), focused learning/discussions, and continued access to resources^25^ including a digital navigator^49^ and funding.^51^ Recommendations regarding how best to address these challenges among YYAs is lacking; however, it is likely that similar approaches would work for this group. As part of our work, an implementation team that included mental health clinicians supported clinical sites with education, troubleshooting, and initial onboarding activities. As the project expands, future implementation approaches should consider providing clinical sites with longer-term support through continuing education and access to training environments within the platform. Future research should identify how best to address the need for ongoing education in YYAs and explore how addressing organizational support at all levels impacts sustainability of the intervention.

Health care provider “buy-in” to dMH interventions has been shown to influence patient uptake,^48^ particularly in the context of an established therapeutic relationship.^27,48^ Degree of provider “buy-in” to digital health has been associated in part with their perceptions of patient/client “buy-in”^52^; our results suggest that a similar association exists among YYAs toward their mental health providers. Alignment of stakeholder views has been recognized in the general literature as a contributing factor to successful implementation,^24,52^ but it has yet to be fully explored in this context. To better understand the implementation factors associated with the *inner setting and implementation process* domains, we explored both YYA and provider perceptions of the others’ willingness to use dMH. Like work conducted in other settings,^53,54^ the misalignment that we identified represented a significant barrier to implementation in our work. It has been suggested that this challenge can be addressed by incorporating both ongoing and iterative feedback into implementation activities,^24,52^ continuing education, and ongoing communication between stakeholders.^24^ Importantly, increasing alignment/changing perceptions may ultimately improve YYA engagement in care and strengthen collaborative relationships; further qualitative research exploring and identifying contributors to this misalignment is needed to support future implementation efforts. Furthermore, research regarding the impact of this on long-term patient and system outcomes following implementation of dMH for YYA mental health care is needed.

### Recommendations for implementation

Several recommendations were developed from this work. First, training regarding the purpose and use of the platform should be ongoing past initial socialization and onboarding. Education should reflect changing clinical practices (e.g. new charting methods/workflows) and include access to training environments within the platform. Second, integration of the platform into clinical settings and client lives should be intentional. For providers, integration should reflect current workflows, duplication of tasks (e.g. charting) should be minimized, and any changes to workflow and/or scheduling (e.g. adding extra time into appointments for review, or restructuring appointment flow) should be explored with providers and management. For YYAs, integration should consider individuals’ devices (e.g. phone make/model), lifestyle, and motivation for using dMH. Finally, feedback from providers and YYAs should be regularly collected and shared back to both groups so that misperceptions and “sticking points” (including differing views on usefulness, treatment goals) can be identified and implementation processes and associated education adjusted accordingly.

### Strengths and limitations

Several limitations must be acknowledged. While respondents represented a variety of clinical settings and population centers across the province, the sample sizes were small. Findings therefore may not be generalizable to settings outside of Alberta. Additionally, these findings reflect the experiences of providers and YYAs who are likely to use/or consider using the platform. As providers were able to choose who to invite to use the platform, the experiences of YYAs with certain characteristics (e.g. more severe mental health concerns; inability to access the platform) were not included.

This work has several strengths. Importantly, it is one of few studies that include YYA experiences and perceptions of the processes underlying implementation of dMH. Additionally, inclusion of both service providers and users allowed us to identify barriers to implementation that had not yet been discussed in the literature. Finally, the breadth of questions asked in the surveys allowed for identification of specific barriers and facilitators regarding both the use of the dMH platform and the implementation approach.

## Conclusion

Our results suggest that providers and YYAs support the implementation of a dMH platform into care across several clinical and community settings. This study identified several factors that could influence the implementation of dMH, including user perceptions of acceptability. Providing continuing and hands-on education for providers and YYAs has the potential to further increase uptake among both groups. Our findings suggest that misalignment between provider and YYA perceptions of each other's willingness to use the platform is an important barrier to consider when developing implementation approaches. Implementation strategies should address these gaps to ensure that dMH interventions are matched to the needs of a variety of potential users.

## Supplemental Material

sj-docx-1-dhj-10.1177_20552076241289179 - Supplemental material for Mental health provider and youth service users’ perspectives regarding implementation of a digital mental health platform for youth: A survey study Supplemental material, sj-docx-1-dhj-10.1177_20552076241289179 for Mental health provider and youth service users’ perspectives regarding implementation of a digital mental health platform for youth: A survey study by Julia Hews-Girard, Katherine Bright, Marianne Barker, Emily M Bassi, Frank Iorfino, Haley M LaMonica, Karen Moskovic, Melanie Fersovitch, Leanne Stamp, Jessica Gondziola, Ian Hickie and Gina Dimitropoulos in DIGITAL HEALTH

sj-docx-2-dhj-10.1177_20552076241289179 - Supplemental material for Mental health provider and youth service users’ perspectives regarding implementation of a digital mental health platform for youth: A survey study Supplemental material, sj-docx-2-dhj-10.1177_20552076241289179 for Mental health provider and youth service users’ perspectives regarding implementation of a digital mental health platform for youth: A survey study by Julia Hews-Girard, Katherine Bright, Marianne Barker, Emily M Bassi, Frank Iorfino, Haley M LaMonica, Karen Moskovic, Melanie Fersovitch, Leanne Stamp, Jessica Gondziola, Ian Hickie and Gina Dimitropoulos in DIGITAL HEALTH

sj-docx-3-dhj-10.1177_20552076241289179 - Supplemental material for Mental health provider and youth service users’ perspectives regarding implementation of a digital mental health platform for youth: A survey study Supplemental material, sj-docx-3-dhj-10.1177_20552076241289179 for Mental health provider and youth service users’ perspectives regarding implementation of a digital mental health platform for youth: A survey study by Julia Hews-Girard, Katherine Bright, Marianne Barker, Emily M Bassi, Frank Iorfino, Haley M LaMonica, Karen Moskovic, Melanie Fersovitch, Leanne Stamp, Jessica Gondziola, Ian Hickie and Gina Dimitropoulos in DIGITAL HEALTH
