# Supplementary material for: Mental health provider and youth service users’ perspectives regarding implementation of a digital mental health platform for youth: A survey study
Source: Digit Health. 2024 Oct 14;10:20552076241289179. doi: 10.1177/20552076241289179 (PMC11483713; doi:10.1177/20552076241289179)
Supplement: sj-docx-1-dhj-10.1177_20552076241289179 - Supplemental material for Mental health provider and youth service users’ perspectives regarding implementation of a digital mental health platform for youth: A survey study [file sj-docx-1-dhj-10.1177_20552076241289179.docx]

**Supplemental Table**

Table 1: Demographics of non-users

|  | Providers (n=52) |  | Youth & Young Adults (n=16) |
| --- | --- | --- | --- |
| Age (years)  <30  30-39  40-49  >50 | 11 (21.2)  13 (25.0)  14 (26.9)  14 (26.9) | Age (years)  > 18  Other^A^ | 12 (75.0)  unable to report |
| Community  Large population center  Medium population center  Small population center | 8 (15.4)  14 (26.9)  29 (55.8) | Community  Large population center  Small population center  other^B^ | 9 (56.3)  5 (31.3)  unable to report |
| Professional Designation  Social Work  Counsellor/Therapist  Other* | 21(40.4)  13 (25.0)  18 (34.6) | Employment Status  Student  Employed | 8 (50.0)  13 (81.3) |
| Place of Work  Specialized Mental Health  School  Other** | 33 (63.5)  13 (25.0)  6 (11.5) | Living Situation  Living with parents  Single | 12 (75.0)  12 (75.0) |
| Years in Role***  < 5 years  > 6  Years in Organization  < 5 years  > 6 years | 37 (71.2)  15 (28.8)  27 (51.9)  25 (48.1) |  |  |

Note: all are n (%) unless otherwise noted; ***13/37 (8.1%) individuals with < 5 years in the role also reported not having direct contact with clients (i.e. just moved into a supervisor position); 19/52 (36.5%) of all individuals reported no direct contact with clients; *other = administrator, psychology, nurse, teacher; **other = PCN, community hub; ^A^other = 15-17/missing/prefer not to say; ^B^other = medium population center/other/missing;
