# Supplementary material for: Mental health provider and youth service users’ perspectives regarding implementation of a digital mental health platform for youth: A survey study
Source: Digit Health. 2024 Oct 14;10:20552076241289179. doi: 10.1177/20552076241289179 (PMC11483713; doi:10.1177/20552076241289179)
Supplement: sj-docx-2-dhj-10.1177_20552076241289179 - Supplemental material for Mental health provider and youth service users’ perspectives regarding implementation of a digital mental health platform for youth: A survey study [file sj-docx-2-dhj-10.1177_20552076241289179.docx]

Supplemental Material

**Mental Health Provider Early Implementation Survey**

**Survey 1.0 - Lead in for the Early Implementation Clinician Survey**

1. **Which of the following best describes you:**
2. I have completed eMental Health training but have not set up my profile for the Innowell platform
3. I have completed the eMental Health training and have set up my profile for the Innowell platform. However, I have not invited any youth to the platform
4. I have completed the eMental Health training, have set up my profile, and have invited youth to the platform. However, I do not have youth who have accepted their platform invitation
5. I have completed eMental Health training, have set up my profile, and have worked with youth on the platform.
6. I have completed the eMH training, have set up my profile, and have onboarded youth onto the platform. However, I have not been able to use the assessment information yet with youth.

**Survey 1.1 - Early Implementation mental health provider Clinician Survey – [These are people that completed training but have not used the platform with youth] [If you answered A/B/C to survey 1.0, skip logic to this section]:**

Even though you have not yet worked with youth on the platform, we greatly value your time in responding to a few questions to help us better understand the barriers to implementation, and how to support mental health providers going forward. Please respond to the following three questions.

1. **Please share the reasons why you have not yet worked on the platform by selecting all that apply.**
   1. I have limited interest in the Innowell platform as a mental health care provider
   2. My organization is not interested in the Innowell platform
   3. Lack of youth interest in the Innowell platform
   4. Lack of family interest in the Innowell platform
   5. Legal Concerns - Liability, responsibility, privacy, and confidentiality
   6. Limited internet connection to access the Innowell platform
   7. Change fatigue in the organization ( i.e., The COVID-19 pandemic, Connect Care, EMR changes, LOCUS, staff turnover/leaves of absence, competing priorities)
   8. Other (Please Specify)
2. **Which of the following might help you integrate the Innowell platform into your work? Please select all that apply.**
3. Improved training about the Innowell platform
4. More information about the value of the Innowell platform in your organization
5. More information about how Innowell can be used to inform treatment planning
6. More information about how Innowell supports the delivery of measurement-based care
7. More information about how Innowell supports therapeutic relationship building
8. Increased research/evaluation in our organization/system
9. Support from organizational leadership to use the Innowell platform
10. Time for mental health providers to use and learn the Innowell platform
11. Clearly defined goals for using the Innowell platform within your site
12. Improved understanding among youth about how to use the Innowell platform
13. Access to reliable Internet/Wi-Fi
14. Access to devices on-site
15. Other (Please Specify)
16. **Please share any additional thoughts on your experience with the eMental Health for Youth and Young Adults in Alberta project or the Innowell platform.** [OPEN TEXT]

**Survey 1.2 Early Implementation Clinician Survey [These are people that have used the platform with youth] [If you answered D to survey 1.0, skip logic to this section]:**

**Intervention Characteristics**

***The Innowell Platform***

1. **Approximately what percentage of youth from your full caseload have you introduced to the Innowell platform within the last few months?**
2. 1-24%
3. 25-49%
4. 50-74%
5. 75-99%
6. 100%
7. **What are the most common reasons for using the Innowell platform? Please select all that apply.**
8. Multi-dimensional assessment purposes
9. Informing treatment planning
10. Outcome monitoring to routinely measure a young person’s progress
11. To provide mental health and substance use care to youth
12. To build a collaborative working relationship with youth
13. To encourage youth to proactively work on their mental health care
14. To improve collaboration with other service providers in the same organization
15. To improve communication with other service providers ***outside*** of the organization
16. Monitor for youth at high risk for suicidality
17. To share resources, such as apps and e-tools with youth
18. Youth interest in the Innowell platform
19. Family interest in the Innowell platform
20. Other (Please specify) **[Skip logic – if other]** What are the most common reasons for using the Innowell platform? [OPEN TEXT]
21. **Approximately how frequently are you and youth reviewing the Innowell platform in your sessions?**
22. Never
23. Less than half of sessions
24. More than half of sessions
25. Every Session
26. **To what extent has the use of the Innowell platform contributed to the following. (Likert Scale – 1 – no increase, 2 – Increased slightly, 3 – Increased somewhat, 4 – Increased moderately, 5 – Increased a lot).**
27. Increased wait times for youth
28. Access to appropriate services for youth
29. Engagement of youth in the assessment process
30. Collaboration with youth regarding treatment planning
31. Collaboration between community sites/services
32. Trust building with the youth
33. Time with youth in session
34. Identification of youth mental health and substance use needs
35. Ensure youth get the right service for their mental health and substance use concerns
36. Efficiency of my mental health practice with youth
37. Resources for youth (i.e., apps and e-tools)
38. Improved youth outcomes
39. Improved management of safety plan
40. Addressing service gaps

***Client Specific***

1. **What criteria do you use to decide which youth you introduce the Innowell platform to?**
   1. Youth that are low risk for mental health and substance use problems
   2. Youth that are high risk for mental health and substance use problems
   3. Only youth that I know very well
   4. All new clients
   5. Nothing - I invite all youth to the platform
   6. Other (Please specify)
2. **Please rate the statements below based on your use of the Innowell platform over the past few months.**

- I am able to offer comprehensive assessments to my clients through the use of the Innowell platform

LIKERT SCALE: 1 – Strongly disagree 2 - Disagree 3 - Neither agree nor disagree 4 – Agree 5 – Strongly agree

- Measurement-based care implemented through the Innowell platform is an important way to monitor my client’s progress while we are working together

LIKERT SCALE: 1 – Strongly disagree 2 - Disagree 3 - Neither agree nor disagree 4 – Agree 5 – Strongly agree

- The Innowell platform helps me provide more comprehensive care for my clients

LIKERT SCALE: 1 – Strongly disagree 2 - Disagree 3 - Neither agree nor disagree 4 – Agree 5 – Strongly agree

- I am able to provide better discharge planning to my clients through the use of the Innowell platform

LIKERT SCALE: 1 – Strongly disagree 2 - Disagree 3 - Neither agree nor disagree 4 – Agree 5 – Strongly agree

**How often have you supported youth with their use of an app on the platform (e.g. walk them through it, answer questions they have about the app)?**

1. Never
2. Not very often
3. Sometimes
4. Fairly often
5. Very often

**Inner Setting**

1. **Please rate the statements below based on the implementation of the Innowell platform over the past few months.**

- This Innowell platform was easily implemented during **intake** of clients into my care

LIKERT SCALE: 1 – Strongly disagree 2 - Disagree 3 - Neither agree nor disagree 4 – Agree 5 – Strongly agree

- This Innowell platform was difficult to implement during **treatment planning** with clients

LIKERT SCALE: 1 – Strongly disagree 2 - Disagree 3 - Neither agree nor disagree 4 – Agree 5 – Strongly agree

- This Innowell platform was easily implemented during the **treatment phase** with clients

LIKERT SCALE: 1 – Strongly disagree 2 - Disagree 3 - Neither agree nor disagree 4 – Agree 5 – Strongly agree

- The Innowell platform was difficult to implement during **case review** with my clients

LIKERT SCALE: 1 – Strongly disagree 2 - Disagree 3 - Neither agree nor disagree 4 – Agree 5 – Strongly agree

1. **What are the barriers to implementing the Innowell platform in your service? Select all that apply.**
2. Lack of understanding about what the platform is and how it’s supposed to be used
3. Doesn’t offer any clinical value
4. Worry that technology poses a potential risk to quality of care provided to clients
5. Lack of support from organizational leadership to use the Innowell platform
6. Lack of youth interest in the Innowell platform
7. Lack of family interest in the Innowell platform
8. Time restraints - Services that are only open a few days per week
9. Capacity restraints
10. Youth not understanding how to use the Innowell platform
11. Lack of personal capacity/interest/awareness
12. Lack of technical support/training
13. Lack of clinical training about the utility of the Innowell platform
14. Privacy and confidentiality concerns with using the Innowell platform
15. Concerns regarding liability/responsibility for the youth in off-hours
16. Research/site consent process (including minors)
17. The Innowell platform does not seem advanced enough to enable clinical areas (i.e., routine outcome monitoring, multi-dimensional assessment)
18. Technological limitations in my practice/service (i.e, Limited access to reliable Internet and devices)
19. The COVID-19 pandemic
20. Legal agreements
21. Other system/site changes (i.e. Connect Care, EMR changes, LOCUS)
22. Staff turnover/leaves of absence
23. Service closure (i.e. school holidays)
24. Access to devices on-site
25. Other (Please Specify)
26. **What might help you implement the Innowell platform? Select all that apply.**
27. Improved training provided by the eMH project team
28. More information about the value of the Innowell platform in our organization/system
29. Support from organizational leadership to use the Innowell platform
30. Time for clinicians to use and learn the Innowell platform
31. More clearly defined project goals for using the Innowell platform with youth
32. Youth understanding how to use the Innowell platform
33. Access to reliable Internet
34. Access to devices on-site
35. Additional service roles or supports (i.e., digital navigators to help youth engagement)
36. Changes to clinical process to better integrate the Innowell platform into workflows and practices
37. Other (Please Specify) **[Skip logic – other]** What might have helped your service better implement the Innowell platform?

**Outer Setting**

1. **Please rate the statements below based on your use of the Innowell platform over the past few months.**

- The Innowell platform meets the needs of my clients

LIKERT SCALE: 1 – Not at all 2 – Very little 3 – Neutral 4 – Somewhat 5 – To a great extent

- I was able to integrate the Innowell platform into my practice

LIKERT SCALE: 1 – Not at all 2 – Very little 3 – Neutral 4 – Somewhat 5 – To a great extent

- Time is a major obstacle for **me** implementing the Innowell platform

LIKERT SCALE: 1 – Strongly disagree 2 - Disagree 3 - Neither agree nor disagree 4 – Agree 5 – Strongly agree

- Time is a major obstacle for my **clients** using the Innowell platform

LIKERT SCALE: 1 – Strongly disagree 2 - Disagree 3 - Neither agree nor disagree 4 – Agree 5 – Strongly agree

- My clients’ severity of mental health/substance use symptoms is a major obstacle for **me** implementing the Innowell platform

LIKERT SCALE: 1 – Strongly disagree 2 - Disagree 3 - Neither agree nor disagree 4 – Agree 5 – Strongly agree

- My clients’ severity of mental health/substance use symptoms is a major obstacle for **their** use of the Innowell platform

LIKERT SCALE: 1 – Strongly disagree 2 - Disagree 3 - Neither agree nor disagree 4 – Agree 5 – Strongly agree

- Technical accessibility issues (lack of technology/lack of reliable Wi-Fi/lack of devices) are a major obstacle for **me** implementing the Innowell platform

LIKERT SCALE: 1 – Strongly disagree 2 - Disagree 3 - Neither agree nor disagree 4 – Agree 5 – Strongly agree

- Technical accessibility issues (lack of technology/lack of reliable Wi-Fi/lack of devices) are a major obstacle for **my clients** using the Innowell platform

LIKERT SCALE: 1 – Strongly disagree 2 - Disagree 3 - Neither agree nor disagree 4 – Agree 5 – Strongly agree

- The resources in the Innowell platform are helpful for my clients to self-manage their mental health and substance use symptoms

LIKERT SCALE: 1 – Strongly disagree 2 - Disagree 3 - Neither agree nor disagree 4 – Agree 5 – Strongly agree

- The apps in the Innowell platform are helpful for my clients to self-manage their mental health and substance use symptoms

LIKERT SCALE: 1 – Strongly disagree 2 - Disagree 3 - Neither agree nor disagree 4 – Agree 5 – Strongly agree

**Implementation Climate**

1. **Please rate the statements below based on your use of the Innowell platform over the past few months.**

- The level of difficulty implementing the Innowell platform into my clinical practice was

LIKERT SCALE: 1 – Very difficult 2 – Difficult 3 – Neutral 4 – Easy 5 – Very easy

- The Innowell platform helps me provide more comprehensive care that is better able to meet the needs of youth served in my organization/clinic

LIKERT SCALE: 1 – Strongly disagree 2 - Disagree 3 - Neither agree nor disagree 4 – Agree 5 – Strongly agree

- What degree of support did you receive for the implementation of the Innowell platform into your clinic care from your **management team?**

LIKERT SCALE: 1 – Strongly opposed 2 – Somewhat opposed 3 – Neutral 4 – Somewhat supported 5 – Strongly supported

- What degree of support did you receive for the implementation of the Innowell platform from your clinic care from your **clinic/organization?**

LIKERT SCALE: 1 – Strongly opposed 2 – Somewhat opposed 3 – Neutral 4 – Somewhat supported 5 – Strongly supported

1. **What has helped you use the Innowell platform since it was implemented in your organization? Select all that apply.**
2. Personal capacity (skill level, knowledge and awareness of the Innowell platform)
3. Professional skill level regarding comfort/confidence with various assessments
4. Technology support (access to Internet and devices)
5. Technology assistance with resolving issues with the Innowell platform
6. Leadership providing time for learning about the Innowell platform
7. Administrative support, such as navigating the Innowell platform
8. Effective communication about how to use the Innowell platform
9. Effective leadership in supporting the team to integrate the Innowell platform
10. Clear expectations of accountability for using the Innowell platform
11. Clear procedures for youth in crisis
12. Access to reliable Internet
13. Implementation lead support (e.g., eMH team member)
14. Project management support (e.g., eMH team member)
15. My team’s previous experience navigating change
16. Other (Please specify) [**Skip logic – if other]** If other, what enables you to use the Innowell platform since it was implemented in your system/organization? [Open text]

**Characteristic of Individual**

1. **Please rate the statements below based on your use of the Innowell platform over the past few months.**

- I believe that the Innowell platform is helpful in engaging youth in mental health services

LIKERT SCALE: 1 – Strongly disagree 2 - Disagree 3 - Neither agree nor disagree 4 – Agree 5 – Strongly agree

- I believe that the Innowell platform helps me to create positive experiences with the youth I work with

LIKERT SCALE: 1 – Strongly disagree 2 - Disagree 3 - Neither agree nor disagree 4 – Agree 5 – Strongly agree

- I believe that the Innowell platform helps build relationships with the youth I work with

LIKERT SCALE: 1 – Strongly disagree 2 - Disagree 3 - Neither agree nor disagree 4 – Agree 5 – Strongly agree

**Process**

1. **Please rate the statements below based on your use of the Innowell platform over the past few months.**

- I felt comfortable implementing the Innowell platform at my service setting

LIKERT SCALE: 1 – Strongly disagree 2 - Disagree 3 - Neither agree nor disagree 4 – Agree 5 – Strongly agree

- My organization uses a strategy of **education** to implement the Innowell platform

LIKERT SCALE: 1 – Strongly disagree 2 - Disagree 3 - Neither agree nor disagree 4 – Agree 5 – Strongly agree

- My organization uses a strategy of **mentoring** to implement the Innowell platform

LIKERT SCALE: 1 – Strongly disagree 2 - Disagree 3 - Neither agree nor disagree 4 – Agree 5 – Strongly agree

- My organization uses a strategy of **training** to implement the Innowell platform

LIKERT SCALE: 1 – Strongly disagree 2 - Disagree 3 - Neither agree nor disagree 4 – Agree 5 – Strongly agree

- My organization has individuals who are responsible for leading implementation of innovation, such as the Innowell platform

LIKERT SCALE: 1 – Strongly disagree 2 - Disagree 3 - Neither agree nor disagree 4 – Agree 5 – Strongly agree

- My clinic/organization has individuals who champion the Innowell platform

LIKERT SCALE: 1 – Strongly disagree 2 - Disagree 3 - Neither agree nor disagree 4 – Agree 5 – Strongly agree

1. **Please share any additional thoughts on your experience with the eMental Health for Youth and Young Adults in Alberta project or the Innowell platform. [Open Text]**
