# Supplementary material for: Mental health provider and youth service users’ perspectives regarding implementation of a digital mental health platform for youth: A survey study
Source: Digit Health. 2024 Oct 14;10:20552076241289179. doi: 10.1177/20552076241289179 (PMC11483713; doi:10.1177/20552076241289179)
Supplement: sj-docx-3-dhj-10.1177_20552076241289179 - Supplemental material for Mental health provider and youth service users’ perspectives regarding implementation of a digital mental health platform for youth: A survey study [file sj-docx-3-dhj-10.1177_20552076241289179.docx]

Supplemental Material

**Youth Early Implementation Survey**

**Section 1: Your Perspective Using the Platform**

1. **We would like to understand your perspectives. Please use this scale to rate your level of agreement or disagreement with the following statements. [Scale from 1 – Strongly**

**disagree, to 5 – strongly agree.]**

1. The Innowell platform is a good addition to the mental health care I receive
2. The Innowell platform is effective in maintaining the security of my information
3. The Innowell platform helps me and my health care provider collaborate on my wellbeing
4. I would refer a friend or family member to a service that offers the Innowell platform
5. The Innowell platform helps me track my mental health in-between sessions
6. The Innowell platform helped me share about my mental health with my mental health care provider (therapist, social worker, school counsellor, etc.)

**Section 2: Use of the Platform, Apps & e-Tools**

We would like to understand your use of the Innowell platform.

1. **On average, how many times (in total) per month do you typically use the Innowell platform?**
2. I do not use the Innowell platform
3. Once a month
4. 2 to 5 times a month
5. 6 to 10 times a month
6. More than 10 times a month
7. Other [Skip logic] If other: Please elaborate (Open Text)
8. **If answered A, then [skip logic] If you did not use the Innowell platform, please share why. Select all that apply.**
   1. I am not interested in using the Innowell platform
   2. The Innowell platform is too hard for me to use
   3. The Innowell platform did not seem helpful for my mental health and wellness
   4. My mental health care provider (Therapist, counsellor, social worker, dr., etc.) is not interested in using the Innowell platform with me
   5. I don’t have enough time to use the Innowell platform
   6. Other (Please specify)

[Skip logic: to next question for youth who do not use the platform]

1. **What would help you take a step to using the Innowell platform? Select all that apply.**
2. If the Innowell platform was easier to use
3. If my mental health provider reviewed how to use the Innowell platform with me
4. If there were more instructions on how to use the Innowell platform (e.g. when to complete questionnaires, what to do with questionnaire results)
5. If my mental health provider seemed more interested in using the Innowell platform with me
6. If I better understood how the Innowell platform could help me

[Skip logic: to next question for youth who **have** used the platform]

1. **When using the Innowell platform, how long do you normally stay logged on each time?**
   1. 0 - 15 minutes
   2. 16 - 30 minutes
   3. 31 - 45 minutes
   4. 46 - 60 minutes
   5. Other [Skip logic] if other: Please elaborate.
2. **When do you usually use the Innowell platform? Select all that apply.**
   1. In session with my mental health care provider (e.g., therapist, counsellor, social worker, dr., psychiatrist, etc.).
   2. In my own time (e.g., at home, at school)
   3. Both in sessions with my mental health provider and on my own time
   4. During time spent with family members
   5. Other (Please specify)

[Skip logic] If other: When do you usually use the platform? (Open text)

1. **What are your most common reasons for using the Innowell platform? Select all that apply.**
   1. Updating my questionnaire results to track my mental health and wellness outcomes
   2. Requesting care from my mental health care provider (e.g., therapist, counsellor, social worker, dr., psychiatrist, etc.).
   3. Viewing my questionnaire results to better understand my mental health and wellness
   4. Accessing options when I am in crisis (e.g., 24/7 phone line)
   5. Accessing mental health information, apps, and e-tools
   6. Other (Please specify)

[Skip logic] If other: What are your reasons for using the platform? (Open Text)

**Section 3: Facilitators and Barriers to your use of the Innowell platform**

1. **We would like to understand the benefits and disadvantages of using the Innowell platform. Please use this scale to rate your level of agreement or disagreement with the following statements. [Scale from 1 – Strongly disagree, to 5 – strongly agree.]**
2. I learned about mental health overall by using the Innowell platform
3. I learned about my own mental health experience by using the Innowell platform
4. My mental health improved by using the Innowell platform
5. I received increased access to supports/services by using the Innowell platform
6. I felt more involved in my mental health care by using the Innowell platform
7. I experienced reduced wait time for services by using the Innowell platform
8. Using the Innowell platform helped my mental health care provider and I shared similar goals for my mental health care
9. I felt that my mental health provider better understood my mental health by using the Innowell platform
10. The Innowell platform data helped me and my provider view changes in my health over time
11. **What did you like *most* about the Innowell platform? [Open text]**
12. **What did you like *least* about the Innowell platform? [Open text]**
13. **Please select any barriers you may have encountered in using the Innowell platform. Select all that apply.**
14. Difficulty with the research consent process
15. Difficulty understanding how the Innowell platform helps my mental health and wellness
16. Not seeing value in using the Innowell platform
17. Difficulty navigating the Innowell platform
18. Forgetting to use the Innowell platform on a regular basis
19. My mental health provider doesn’t seem that interested in using the Innowell platform with me
20. Difficulty understanding the questions on the Innowell platform
21. Lack of access to devices
22. Lack of access to reliable internet
23. Other [skip logic: If other - please specify.
24. **Is there anything that would have helped you use the platform more? Select all that apply.**
    1. Better training from my mental health provider about how to use the Innowell platform
    2. Access to reliable internet
    3. Access to reliable devices
    4. Increased communication about the Innowell platform by my mental health care provider
    5. Understanding the relationship between the Innowell platform and my mental health and wellness
    6. Text or email reminders
    7. Other [skip logic]if other, please specify [open text].

**Section 4: Apps & e-tools**

We would like to understand your use of apps and e-tools. Please answer the following questions.

1. **How often do you use apps or e-tools on the Innowell platform per month?**
2. Never
3. Not very often
4. Occasionally
5. Fairly often
6. Very often
7. **Which apps did you try, if any? Select all that apply.**

- Mindshift
- Smiling Mind
- SuperBetter
- Insight Timer
- Mental Health Line
- CBT-i Coach
- Sleep Cycle
- Togetherall
- Now Matters Now
- Drinks Meter
- Habit-bull: Daily Goal Tracker
- Saying When
- Checking Your Drinking 3.0
- Woebot
- Sanvello
- Healthy minds
- Recovery Record
- Rise Up + Recover
- Apart of Me
- Nike Training Club
- Zombies, Run!
- ParticipACTION
- MyFitness Pal
- MoodMission
- PTSD Coach Canada
- OCD Challenge
- Early Psychosis Intervention
- Mindshine
- eQuoo
- Calm Harm
- My Study Life
- MyQuitBuddy
- Break It Off

1. **Please use this scale to rate your level of agreement or disagreement with the following statements about the apps and e-tools portion of the Innowell platform from strongly disagree to strongly agree. If the answer is not applicable for you, please mark N/A.**
2. I am interested in using the apps or e-tools
3. I would like to use apps or e-tools with my mental health provider
4. The apps or e-tools are easy for me to use
5. The apps or e-tools are helpful to my mental health
6. I am interested in using the apps or e-tools, but lacking in motivation

**Section 5: Questionnaires**

We would like to understand your experience responding to the questionnaires. These questionnaires include answering all the questions when you first joined the platform, along with any other times you may have updated your responses.

1. **How often do you update your responses to the questionnaires?**
   1. Never
   2. Not very often
   3. Every session with my mental health provider
   4. As needed
   5. More than every session
2. **Please use this scale to rate your experience responding to the questionnaires from 1 – Strongly disagree, to 5 – strongly agree. If the answer is not applicable for you, please mark N/A.**
3. Going through the questionnaires was easy for me
4. I am interested in using the questionnaires on the Innowell platform
5. Viewing my questionnaire results immediately is helpful for me
6. Viewing my questionnaire results was upsetting to me
7. Reviewing my questionnaire results with my mental health provider is helpful for me
8. I feel motivated to use the questionnaires
9. I have lots of time to update the questionnaires
10. Tracking how I was doing over time by using the Innowell platform is helpful for me

**Section 6: Experience Using Platform**

1. **We would like to understand your experience using the Innowell platform. Please use this scale to rate your level of agreement or disagreement with the following statements. [Scale from 1 – Strongly disagree, to 5 – strongly agree.] If the answer is not applicable for you, please mark N/A.**
2. I feel that I understand how the Innowell platform works
3. I felt like I had a choice regarding what questions to answer on the Innowell platform
4. I did **not** feel pressured to share information I was uncomfortable with sharing
5. When I received a pop-up message that I scored high on a questionnaire on the Innowell Platform, I did **not** find it overwhelming
6. I have a good idea of when I should use the Innowell platform
7. **Has your mental health provider ever followed up with you outside of a session about a high or concerning score on one of your Innowell questionnaires?**
8. Yes
9. No
10. **[Skip logic - YES]: If yes, go to the following. When your mental health provider followed up with you outside of a session about a high or concerning score on one of your Innowell questionnaires, what was your experience? Please select all that apply.**
11. I was surprised
12. I felt supported
13. I appreciated the follow-up
14. It helped me manage my thoughts and behaviours
15. I felt annoyed
16. Other [Skip logic] If other, please describe_______________
17. **Is there anything you would like to add? _____________________________**
